# A systematic review on the effect of silver diamine fluoride for management of dental caries in permanent teeth

**DOI:** 10.1002/cre2.716

**Published:** 2023-02-23

**Authors:** Alvin Mungur, Haoran Chen, Saroash Shahid, Aylin Baysan

**Affiliations:** ^1^ Institute of Dentistry, Barts and The London School of Medicine and Dentistry Queen Mary University of London London UK

**Keywords:** arrest/reversal, dental caries, permanent teeth, silver diamine fluoride

## Abstract

**Objectives:**

The objective of this review is to assess the available literature systematically related to the effect of silver diamine fluoride (SDF) for the management of occlusal and root carious lesions in permanent teeth regardless of age.

**Materials and Methods:**

This systematic review was conducted according to the Cochrane Handbook for Systematic Reviews of Interventions and Preferred Reporting Items for Systematic Review and Meta‐Analyses statement. A literature search was performed using PubMed/MEDLINE, Cochrane Central Register of Controlled Trials, Web of Science, DOAJ, and Open Gray with no language restrictions up to December 2022. Three reviewers critically assessed the studies for eligibility. Any disputes between the reviewers were handled by a fourth independent reviewer. The quality assessment and data extraction of the studies were performed.

**Results:**

A total of 2176 studies were screened. The titles and abstracts of the studies were then reviewed (*n* = 346), and 52 studies met the search criteria. Following the full‐text review, 11 studies investigated the effect of SDF against other treatments such as chlorhexidine, sodium fluoride, ammonium bifluoride, tricalcium silicate paste, casein phosphopeptide amorphous calcium phosphate, glass ionomer cement (GIC) combined with fluoride varnish, resin‐modified GIC, and atraumatic restorative treatment were assessed.

**Conclusions:**

Within the limitations of this review, the use of SDF is promising with high preventative fractions in permanent teeth of children and older populations when compared to other topical applications such as dental varnish containing sodium fluoride.

## 1 INTRODUCTION

Dental caries is still an important public health problem. The World Health Organisation (WHO) indicated that this disease affects not only 60%–90% of schoolchildren but also the majority of adults. This noncommunicable disease is one of the main causes of loss of natural teeth in the aging population (Petersen et al., 2010).

Silver compounds such as silver nitrate have been used in dentistry due to their antimicrobial properties for many decades (Horst et al., 2017; Peng et al., 2012). The cariostatic effect of silver nitrate is believed to be related to the formation of calcified or sclerotic dentine (Stebbins, 1891). Although there was a decline in the use of silver compounds, these compounds have been reintroduced relatively recently due to their low cost and ease of application (Gao et al., 2018).

Silver diamine fluoride [SDF, Ag(NH_3_)_2_F] is an alkaline (pH~8–9), and colorless topical agent comprising silver and fluoride (Shah et al., 2013). SDF combines the remineralizing effect of fluoride with the antimicrobial effect of silver, which makes SDF treatment effective in controlling carious lesions in comparison to other fluoride treatments such as sodium fluoride varnish (Shah et al., 2013). The mechanism of action is that silver ions (Ag^+^) are reported to have antimicrobial effects, and metallic silver (Ag or Ag°) which is relatively inert. The metallic silver can interact with moisture in an oral environment and would release silver ions (Peng et al., 2012). These ions have been suggested to provide three main antimicrobial effects: the destruction of cell wall structure; denaturation of the cytoplasmic enzyme, and inhibition of microbic DNA replication. SDF penetrates the enamel to a depth of up to 25 µm, and approximately 2–3 times more fluoride could be retained in comparison to sodium fluoridemonophosphate, sodium fluoride, or stannous fluoride. Furthermore, the storage period of SDF is longer than that of AgF since Ag^+^ in SDF can be stabilized by forming a silver‐diamine complex, [Ag(NH_3_)_2_F] (Liu et al., 2012).

The concentration of the most commonly used SDF was 38%, which has up to 44,800 ppm fluoride. The remineralization action of SDF on dental caries could be attributed to its high concentration of fluoride, alkaline property (pH = 8–9) and the presence of silver. SDF contains diamine groups, which might enable the formation of NH_4_OH (ammonium hydroxide) and would potentially promote the optimum required pH and conditions for the mineral formation and enhance antibacterial action. The addition of diamine groups would then stabilize silver ions in AgNO_3_ (Sliver nitrate) solution, forming silver diamine nitrate (SDN), which is expected to enhance the mineral precipitation (Horst et al., 2017; Peng et al., 2012). SDF [Ag(NH_3_)_2_F] reacts with hydroxyapatite (HA) to release calcium fluoride (CaF_2_) and silver phosphate (Ag_3_PO_4_), which arrest carious lesions.

SDF has therefore been regarded as an efficient, affordable, and safe cariostatic agent, therefore its application in dental caries management complies with the concept of Minimally invasive dentistry (MID) (Frencken et al., 2012). Previously, systematic reviews assessed the SDF effect on deciduous teeth and also for the treatment of root caries, specifically for the older population only. This current systematic review focused on permanent teeth both in adults and children regardless of age restrictions. Therefore, the aim of this systematic review was to assess the literature on the effects of SDF for the treatment of dental caries in permanent teeth (adults and children).

### 1.1 Methodology

**Figure 1:**
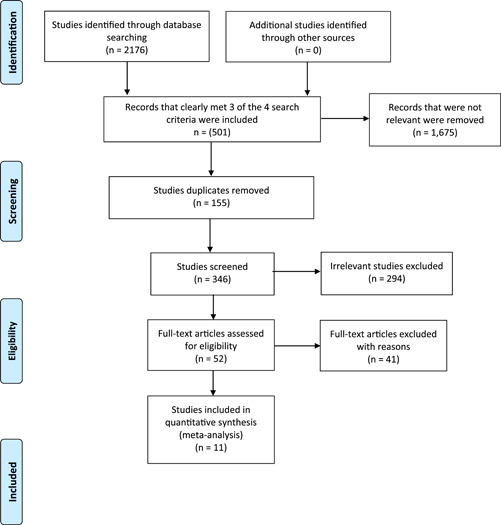
Preferred Reporting Items for Systematic Review and Meta‐Analyses (PRISMA) flow diagram.

This review was conducted according to the guidelines of the Cochrane Handbook for Systematic Reviews of Interventions and Preferred Reporting Items for Systematic Review and Meta‐Analyses (PRISMA) statement (Figure 1). This systematic review followed the four‐phase diagram of the PRISMA (Moher et al., 2009). The PICOS framework was used (Table 1) to formulate the following research question “Is there a difference in the efficacy of silver diamine fluoride in the treatment of dental caries in comparison to other minimally invasive approaches in permanent teeth?” The study protocol was registered with the PROSPERO international prospective register of systematic reviews.

**Table 1:** PICO strategy for the use of silver diamine fluoride (SDF) on permanent teeth.

Population	Permanent teeth with dental caries
Intervention	The use of SDF for the treatment of dental caries
Comparison	Current minimally invasive techniques for the treatment of dental caries
Outcome	Remineralisation/arrest/reversal of dental caries

### 1.2 Literature search strategy

A comprehensive search was carried out in Medline via Ovid, Scopus, WOS, LILIACS, and Embase. Cochrane Central Register of Controlled Trials and Web of Science to identify studies without language restrictions up to December 2022. Searches in the ClinicalTrials.gov database and the references of the included studies (cross‐referencing), were also conducted. Search on Gray literature was performed using Google, Greylit, and OpenGrey. Medical Subject Headings (MeSH) terms, keywords, and other free terms related to the PICO question (Table 1) were used with Boolean operators (OR, AND) to combine searches. The same keywords were used for all search platforms, following the syntax rules of each database, and the search terms were modified for each database (Table 2).

**Table 2:** Treatment of root caries with silver diamine fluoride (SDF) in adults.

Author, country and year	Study duration (months)	Participant numbers, dropout %, and Month of recording	Intervention (Application at each interval)	Comparison (Application at each interval)	Outcome				
					Number of root surfaces either with new carious lesions or restorations			*p*‐value for comparison against control using analysis of variance one‐way test (statistical significance Y/N)	Preventative fraction (%)
Tan et al., Hong Kong, 2010	36	*N*_0_ = 306 *N*_1_ = 247 (19.3%) [12 months] *N*_2_ = 227 (25.8%) [24 months] *N*_3_ = 203 (33.7%) [36 months]	OHI (3 months) OHI + CHX (3 months) OHI + NaF varnish (3 months) OHI + 38% SDF varnish (12 months)	OHI with the application of water (12 months)	12 months	24 months	36 months	‐	‐
					OHI + water = 1.5 (0.2)	OHI + water = 2.0 (0.3)	OHI + water = 2.5 (0.5)	‐	45
					OHI + CHX = 1.0 (0.2)	OHI + CHX = 1.0 (0.3)	OHI + CHX = 1.1 (0.2)	*p* < 0.01 (Y)	57
					OHI + NaF = 0.8 (0.2)	OHI + NaF = 0.9 (0.2)	*p* < .01 (Y)	64
					OHI + 38% SDF = 0.4 (0.1)	OHI + 38% SDF = 0.7 (0.2)	OHI + 38% SDF = 0.7 (0.2)	*p* < .01 (Y)	71
Zhang et al., Hong Kong, 2013	24	*N*_0_ = 266 *N*_1_ = 227 (14.7%) (24 months)	OHI + 38% SDF (12 months) OHI + 38% SDF (12 months) + OHE (6 months)	OHI with the application of water (12 months)	24 months			‐	‐
					OHI + water = 1.33			‐	4
					OHI + 38% SDF = 1.00			*p* < .05 (Y)	28
					OHI + 38% SDF + OHE = 0.70			*p* < .05 (Y)	33
Li et al., Hong Kong, 2017	30	*N*_0_ = 323 *N*_1_ = 297 (8.0%) [12 months] *N*_2_ = 258 (20.1%) [24 months] N_3_ = 257 (20.4%) [30 months]	OHI + 38% SDF (12 months) OHI + 38% SDF + KI (12 months)	OHI with the application of tonic water (12 months)	12 months	24 months	30 months	‐	‐
					OHI + tonic water = 0.5 (0.1)	OHI + tonic water = 0.9 (0.1)	OHI + tonic water = 1.1 (0.2)	‐	45
					OHI + 38% SDF = 0.2 (0.1)	OHI + 38% SDF = 0.4 (0.1)	OHI + 38% SDF = 0.4 (0.1)	*p* < .001 (Y)	62
					OHI + 38% SDF + KI = 0.2 (0.1)	OHI + 38% SDF + KI = 0.4 (0.1)	OHI + 38% SDF + KI = 0.5 (0.1)	*p* < .001 (Y)	52

*Note*: “‐” indicates information is unobtainable. Abbreviations: CHX, chlorhexidine; KI, potassium iodide; NaF, sodium fluoride; OHE, oral health education programme, limited to 30 min including initiation, assessment, negotiation, commitment and evaluation; OHI, oral health instructions.

There were four main concepts used for the literature search strategy (Supporting Information: Table S1). The first concept was the use of SDF in clinical studies. The second concept was dental caries, where the outcome was assessing any positive effect, such as remineralization or the limitation of caries progression. The third concept was on permanent teeth in different age groups, such as adults and schoolchildren. The fourth concept was also related to the specific dental tissues; root or tooth types such as first permanent molars. Systematic reviews, laboratory‐based/in vitro studies, and case studies were excluded. Studies on primary teeth only with the use of SDF were excluded.

### 1.3 Inclusion and exclusion criteria

The following criteria were followed to include the studies:

- Type of study: Randomized control trials, cohort studies.
- Population: Children, adolescents, and adults, including the aging population.
- Dentition: Permanent teeth.
- Treatment outcomes: Reversal/Arrest of dental caries.
- Language: All languages.

The following studies were excluded:

- Type of study: Case report, in vitro study, laboratory‐based studies, meta‐analysis, systematic reviews, article comments, narrative reviews, studies excluding the use of SDF, studies excluding the use of dental varnish containing fluoride.
- Population: Infants with deciduous teeth and animals.
- Dentition: Primary dentition only.

### 1.4 Data extraction and quality assessment

Literature search results have been de‐duplicated by using EndNote X7 software (Thomson Reuters). Studies have initially been screened based on title, and abstract according to the scope and publication type (i.e., reviews, comments, letters, or abstracts). Three reviewers independently selected the retrieved studies by examining titles and abstracts. The full texts have been accessed when it is not possible to judge the studies by title and abstract. Any discrepancies among authors/reviewers were resolved after careful discussion by the fourth author.

The data were extracted from each included study by two independent reviewers for study identification number, authors, study design, sample size, test tooth, type of dental application, method of analysis, method of outcome assessment, follow‐up, and author's conclusions to create a table of evidence.

### 1.5 Risk of bias assessment

The evaluation was based on the description of the following parameters for the quality assessment of the study: sample size calculation, teeth randomization, standardization of procedures, application by a single operator, blinding of the observer, and statistical analyses carried out. If the authors reported the parameter, the article had a Y (yes) for that specific parameter; if it was not possible to find the information, the article received an N (no). The studies that reported 1–3 items were classified as high risk of bias, 4–6 as medium risk, and 7–8 as low risk. The assessment was carried out by three reviewers, then any disagreements were resolved by discussion and followed up with a consensus.

## 2 RESULTS

The literature was searched through a number of databases (PubMed, Mendeley, and Endnote). The search strategy was also applied to the search engine Google Scholar to include additional studies.

During the first stage of the title and abstract search, studies (*n* = 1675) that failed to meet three of the four search criteria were removed. The next stages of the process were assessed by two reviewers. Any disagreements were taken to be discussed with the fourth reviewer. A number of studies were removed as they were duplicates (*n* = 155). From the remaining papers (*n* = 346), an in‐depth assessment of the title and abstract was undertaken. Papers deemed to be irrelevant by both reviewers were immediately removed (*n* = 294). Five studies were a cause for debate, however, after discussion with the fourth reviewer, these studies were also excluded.

A total of 52 studies were deemed as relevant to review the full texts. 41 publications were then excluded, as either the results were not presented, or the data provided were insufficient to be included. Seventeen studies presented primary teeth or failed to specify the permanent teeth. Twelve studies of clinical trials have not been completed yet. The outcomes of four studies were related to parental acceptability and child comfortability of SDF, hypersensitivity, and cost‐effectiveness of the SDF. As a result, 11 studies matched all the search criteria, were deemed as relevant by all three reviewers, and had sufficient data that could be analyzed and compared (Table 3a, 3b).

**Table 3a:** Treatment of dental caries with silver diamine fluoride (SDF) in adults.

Author, country and year	Study duration (months)	Participant numbers, dropout percentage and month of recording	Intervention (application every interval)	Comparison (application every interval)	Outcome										
					Number of root surfaces either with new carious lesions or restorations									*p*‐value for comparison against control using ANOVA one‐way test (statistical significance Y/N)	Preventative fraction (%)
Tan et al., Hong Kong, 2010	36	*N*_0_ = 306 *N*_1_ = 247 (19.3%) [12 months] *N*_2_ = 227 (25.8%) [24 months] *N*_3_ = 203 (33.7%) [36 months]	OHI (3 months) OHI + CHX (3 months) OHI + NaF varnish (3 months) OHI + 38% SDF varnish (12 months)	OHI with the application of water (12 months)	12 months		24 months			36 months				‐	‐
					OHI + water = 1.5 (0.2)		OHI + water = 2.0 (0.3)			OHI + water = 2.5 (0.5)				‐	45
					OHI + CHX = 1.0 (0.2)		OHI + CHX = 1.0 (0.3)			OHI + CHX = 1.1 (0.2)				*p* < .01 (Y)	57
					OHI + NaF = 0.8 (0.2)		OHI + NaF = 0.9 (0.2)			OHI + NaF = 0.9 (0.3)				*p* < .01 (Y)	64
					OHI + 38% SDF = 0.4 (0.1)		OHI + 38% SDF = 0.7 (0.2)			OHI + 38% SDF = 0.7 (0.2)				*p* < .01 (Y)	71
Zhang et al., Hong Kong, 2013	24	*N*_0_ = 266 *N*_1_ = 227 (14.7%) [24 months]	OHI + 38% SDF (12 months) OHI + 38% SDF (12 months) + OHE (6 months)	OHI with the application of water (12 months)	24 months									‐	‐
					OHI + water = 1.33									‐	4
					OHI + 38% SDF = 1.00									*p* < .05 (Y)	28
					OHI + 38% SDF + OHE = 0.70									*p* < .05 (Y)	33
Hamdi et al., Egypt, 2022,	24	*N*_0_ = 45 (92 teeth) *N*_1_ = 45 (92 teeth) (0%) [3 months] N_2_ = 45 (*9*2 teeth) (0%) [6 months] *N*_3_ = 45 (92 teeth) (0%) [12 months] *N*_4_ = 45 (92 teeth) (0%) [24 months]	TCS	Group 1: SDF + KI Group 2: CPP‐ACP	Visual scores by DIAGNOdent									‐	Information unavailable
					3 months		6 months		12 months			24 months			
					Score 1	Score 2	Score 1	Score 2	Score 1		Score 2	Score 1	Score 2		
					TCS = 18 (60%)	TCS = 12 (40%)	TCS = 28 (93.3%)	TCS = 2 (6.7%)	TCS = 30 (100%)		TCS = 0	TCS = 30 (100%)	TCS = 0	*p* < .001	
					SDF + KI = 1 (3.2%)	SDF + KI = 30 (96.8%)	SDF + KI = 3 (9.7%)	SDF + KI = 28 (90.3%)	SDF + KI = 5 (16.1%)		SDF + KI = 26 (83.9%)	SDF + KI = 17 (54.8%)	SDF + KI = 14 (45.2%)	*p* < .001	
					CPP‐ACP = 12 (38.7%)	CPP‐ACP = 19 (61.3%)	CPP‐ACP = 29 (93.5%)	CPP‐ACP = 2 (6.5%)	CPP‐ACP = 30 (96.8%)		CPP‐ACP = 1 (3.2%)	CPP‐ACP = 31 (100%)	CPP‐ACP = 0	*p* < .001	
Li et al., Hong Kong, 2017	30	*N*_0_ = 323 *N*_1_ = 297 (8.0%) [12 months] *N*_2_ = 258 (20.1%) [24 months] *N*_3_ = 257 (20.4%) [30 months]	OHI + 38% SDF (12 months) OHI + 38% SDF + KI (12 months)	OHI with the application of tonic water (12 months)	12 months		24 months				30 months			‐	‐
					OHI + tonic water = .5 (0.1)		OHI + tonic water = .9 (0.1)				OHI + tonic water = 1.1 (0.2)			‐	45
					OHI + 38% SDF = 0.2 (0.1)		OHI + 38% SDF = 0.4 (0.1)				OHI + 38% SDF = 0.4 (0.1)			*p* < .001 (Y)	62
					OHI + 38% SDF + KI = 0.2 (0.1)		OHI + 38% SDF + KI = 0.4 (0.1)				OHI + 38% SDF + KI = 0.5 (0.1)			*p* < .001 (Y)	52

*Note*: “‐” indicates information is unobtainable. Abbreviations: CHX, chlorhexidine; KI, potassium iodide; NaF, sodium fluoride; OHE, oral health education programme, limited to 30 min including initiation, assessment, negotiation, commitment and evaluation; OHI, oral health instructions.

**Table 3b:** Treatment with silver diamine fluoride (SDF) for permanent molars in children.

Author, country, year, and tooth structures	Duration (months)	Participant numbers, dropout percentage, and month of recording	Intervention (application at every interval)	Comparison (application at every interval)	Outcome														
					Number of surfaces with new carious lesions or fillings							*p*‐value for comparison against control (statistical significance Y/N)						Preventative fraction (%)	
Mauro et al., Argentina, 2004, permanent first molar	12	*N* = 141 [12 months]	Group 1: 2.26% ammonium bifluoride (one‐time) Group 2: 38% SDF (one‐time) Group 3: 5% NaF (one‐time)	Information unavailable	Information unavailable							Information unavailable						Group 1: 56 Group 2: 57 Group 3: 47	
Llodra et al., 2005, permanent first molar	36	*N*_0_ = 452 *N*_1_ = 373 [36 months] (17.5%)	Group 1: 38% SDF (once every 6 months)	No treatment	Active caries				Inactive caries			Active caries			Inactive caries			‐	
					Group 1: 0.4 (0.1) No treatment: 1.1 (0.2)				Group 1: 0.3 (0.1) No treatment: 0.1 (0)			Group 1: *p* < .001 (Y)			Group 1: *p* < .05 (Y)			Group 1: 72	
Monse et al., Philippines, 2012, permanent first molar	18	*N*_0_ = 1016 *N*_1_ = 704 (30.7%) [18 months]	Group 1: 38% SDF (one‐time) [no programme] Group 2: ART (one‐time) [no programme] Group 3: No treatment [brushing programme^a^] Group 4: 38% SDF (one‐time) [brushing programme] Group 5: ART (one‐time) [brushing programme]	No treatment [no programme]	Group 1: 0.12 Group 2: 0.06 Group 3: 0.08 Group 4: 0.09 Group 5: 0.01 No treatment [no programme]: 0.17							Group 3			No treatment [no programme]			Information unavailable	
												Group 1: *p* > .05 (N) Group 2: *p* < .001 (Y)			Group 4: *p* > .05 (N) Group 5: *p* < .01 (Y)				
Liu et al., Hong Kong, 2012, permanent molars (dentine and fissure)	24	*N*_0_ = 501 *N*_1_ = 485 (3.2%) [24 months]	Group 1: resin sealant (one‐time) Group 2: 5% NaF (6 months) Group 3: 38% SDF (12 months)	Water (12 months)	Tooth					Fissure site		Tooth			Fissure site			Tooth	Fissure site
					Group 1: 3.0 Group 2: 4.4 Group 3: 4.4 Water: 7.4					Group 1: 1.6 Group 2: 2.4 Group 3: 2.2 Water: 4.6		Group 1: *p* < .04 (Y) Group 2: *p* < .04 (Y) Group 3: *p* < .04 (Y)			Group 1: *p* < .002 (Y) Group 2: *p* < .002 (Y) Group 3: *p* < .002 (Y)			Group 1: 60 Group 2: 41 Group 3: 41	Group 1: 65 Group 2: 48 Group 3: 52
Mendiratta et al., India, 2021, permanent posterior teeth	6	*N*_0_ = 82 *N*_1_ = 69, (16.0%) number of teeth = 182 [6 months]	Group 1: 38% SDF	GIC with 5% F	Caries arrest rate										0.405				Group 1 over GIC with 5% F in caries preventive fiction: 45%
					Group 1: 86 (94.5%) GIC with 5% F: 82 (90.1%)														
Baraka et al., Egypt, 2022, permanent molars	12	N_0_ = 49 (108 molars) *N*_1_ = 33 (69 molars) (32.7%) (36.1%) [12 months]	Group 1: 38% SDF + KI Group 2: 38% SDF	RMGIC	Secondary caries														Information unavailable
					3 months					38% SDF + KI = 0(0)			38% SDF = 0(0)		RMGIC = 0(0)		N/A		
					6 months					38% SDF + KI = 0(0)			38% SDF = 0(0)		RMGIC = 0(0)		N/A		
					12 months					38% SDF + KI = 2 (8%)			38% SDF = 0(0)		RMGIC = 0(0)		*p* = .63		
Satyarup et al., India, 2022, permanent molar	9	*N*_0_ = 190 lesions *N*_1_ = 180 lesions (5.3%) [9 months]	Group 1: 38% SDF	ART	Score 1—The restoration intact, covering all pits and fissures		Score 2—The restoration partially lost, and the tooth is sound			Score 3—The restoration partially lost, the tooth is carious			Score 4—The restoration completely lost, the tooth is sound		Score 5—The restoration completely lost, the tooth is carious		*p* = .004		Information unavailable
					Group 1 = 53 (58.9%)	ART = 43 (47.8%)	Group 1 = 19 (21.1%)	ART = 18 (20.0%)		Group 1 = 5 (5.6%)	ART = 15 (16.7%)		Group 1 = 13 (14.4%)	ART = 6 (6.7%)	Group 1 = 0 (0.0)	ART = 8 (8.9%)			

Abbreviations: ART, atraumatic restorative treatment; GIC, glass ionomer cement; NaF, sodium fluoride; RMGIC, resin‐modified glass ionomer cement.

^a^ Brushing programme—The children were involved in a daily toothbrushing programme at school, which included the use of 1450 ppm fluoridated toothpaste.

SDF had the greatest preventative fraction and lowest number of new carious lesions compared to other topical applications.

### 2.1 Statistical analysis

The results of SDF were statistically significant for all studies that posted *p*‐values except for one. There were noticeable differences in the duration of the studies, frequency of application, and method of assessments within each study. The statistical data also varied since some results were given in percentages while some presented either means and standard deviations or provided means of the results alone. Therefore, the included studies were found to be incomparable and too limiting to perform further statistical analyses, including meta‐analysis.

### 2.2 Comparison of the studies

Tan et al. (2010) compared the SDF application and oral health education (OHE) with the control groups that is, OHE only, dental varnishes either containing sodium fluoride or chlorhexidine with OHE and reported an interesting pattern throughout the duration of the study using the analysis of variance (ANOVA) one‐way statistical test (*p* < .05). At the end of the 36‐month study, the number of new carious lesions or restorations on root surfaces was 0.7. This means that even though after the first 12 months, there were only 0.4 new carious lesions or restorations on root surfaces, this increased to be at a consistent rate of 0.7. It should be noted that the control group (OHE + water) presented with more carious lesions or restorations on root surfaces annually. These authors also reported the effect of treatment with chlorhexidine and sodium fluoride. Both treatments had similar *p*‐values to SDF (<0.01) with significant results. However, treatment with SDF had a greater preventative fraction (71%) in comparison to both chlorhexidine (57%) and sodium fluoride (64%). This would indicate that SDF was an effective treatment for arresting root caries in older populations compared to chlorhexidine and/or sodium fluoride.

Zhang et al. (2013) reported that SDF provided significant results for the treatment of root caries (*p* < .05). The treatment of SDF provided a preventative fraction of 28%. The treatment of SDF with Oral Health Education provided a greater preventative fraction of 33%. The difference might only be 5%. This was a clear indication that how education might not just be informative but also would potentially provide a clinical benefit.

The effect of SDF was variable for the treatment of dental caries in first permanent molars in children. Monse et al. (2012) reported insignificant results following the use of SDF either with or without toothbrushing on first permanent molars (*p* > .05). The risk of bias for incomplete outcome data, balanced groups at baseline, measurement reliability, and other bias was unclear. The locations of the two chosen regions, the reason for the number of schools in each region, and the reasoning for having two separate controls to test a number of variables were not explained within the study clearly. The ambiguity failed to give reasons to exclude the results, however, the reliability of the results was in question.

The remaining studies recorded SDF as having significant results (*p* < .05) when compared to the control groups for the treatment of dental caries in first permanent molars in children (Table 3a, 3b). Llodra et al. (2005) assessed the effect of SDF only every 6 months rather than every year. Another unique aspect of this study was the inclusion of new active and inactive carious lesions. The treatment using the SDF had 0.4 new active carious lesions and 0.3 inactive carious. Comparing this with the control (1.1 active carious lesions and 0.1 inactive lesions) showed that the use of SDF might not only arrest caries progression but also inactivate present carious lesions. These authors showed the greatest preventative fraction of 72%. However, this data failed to match the other studies. This was due to the 6‐monthly application of the SDF.

Mauro et al. (2004) reported that the preventative fractions of ammonium bifluoride (56%) and SDF (57%) were almost identical. This would indicate that ammonium bifluoride has similar efficacy in the treatment of dental caries in permanent first molars in children. The use of sodium fluoride had a slightly lower preventative fraction of 47%. This demonstrates the correlation with Tan et al. in comparison to the preventative fraction of SDF (71%) against sodium fluoride (64%). It should be noted that even though Mauro et al. (2004) assessed the effect of SDF on permanent teeth in children, while Tan et al. (2010) investigated the same effect on permanent teeth in older adults. Interestingly, the trend was unchanged.

Liu et al. (2012) investigated the treatment of dental caries on permanent teeth in children with the use of SDF and reported significant results both on the tooth (*p* < .04) as well as at the fissures (*p* < .02). This study reported the preventative fractions for SDF and sodium fluoride to be far more similar than either Tan et al. (2010) or Mauro et al. (2004). Liu et al. (2012) also stated that the participants in the sodium fluoride treatment group were specifically asked not to eat for half an hour after application, which might have had an effect on the results. Another unique aspect of this study was that the application of dental varnish containing sodium fluoride was applied twice a year, while the SDF application was carried out once a year in comparison to Tan et al. (2010) and Mauro et al. (2004).

Recently, Hamdi et al. (2022) reported the remineralization effect of tricalcium silicate (TCS) paste, silver diamine fluoride with potassium iodide (SDF‐KI), and casein phosphopeptide amorphous calcium phosphate (CPP‐ACP) for early enamel lesions on permanent molar teeth (*n* = 92 in 45 participants). The use of SDF‐KI arrested 54.8% of early enamel carious lesions when compared to the TCS and CPP‐ACP groups (100%) for a period of 24 months (*p* < .001). However, SDF‐KI was applied annually only, while the CPP‐ACP and TCS applications were twice a day throughout the study. There is no consensus with regard to the number of applications of SDF on dental caries. It should be noted that the significant remineralization effect of SDF‐KI presented on early enamel lesions following the second application at 24 months.

Interestingly, Mendiratta et al. (2021) compared the 38% SDF application alone with glass ionomer cement (GIC) and fluoride varnish in arresting dental caries that is, Nyvad Score 2/3 in permanent posterior teeth in disabled individuals (*n* = 82). The majority of the study participants were males (79.2%) with being between 11 and 15 years old. It was reported that the caries arrest rate was 94.5% with the SDF while 90.1% with the GIC and fluoride varnish (*p* = .405). In addition, the caries preventive fraction of SDF over GIC with fluoride varnish was 45%. The arrest rate for the SDF group for a period of 6 months was high when compared to the previous studies conducted by Llodra et al. (2005) (65%), and Mauro et al. (2004) (57%).

Subsequently, Baraka et al. (2022) indicated that there were no significant differences (*p* = .26) with regard to secondary caries prevention, pain, and maintaining pulpal health in young permanent first molars with deep occlusal carious lesions by the employment of either resin‐modified GIC (RMGIC), 38% of SDF or SDF plus KI as indirect pulp capping materials followed by resin‐based composite restorations. All restorations were retained successfully without any symptoms of pulpal and apical pathology throughout the study, except for one in the SDF plus KI at 6 months and one in the SDF alone groups after 12 months. Both teeth required root canal therapy. In addition, there were significant differences in restoration color, marginal staining, and luster (*p* = .03). The color and luster of the restorations were found to be favorable for the RMGIC in comparison to both SDF groups. SDI‐KI group presented with a temporary reduction of the black staining effect related to SDF, however, restorations in this group seemed to darken over time.

Satyarup et al. (2022) investigated the effectiveness of 38% SDF and atraumatic restorative treatment (ART) for treating dental caries of permanent molars in children for a period of 9 months. The SDF treatment group presented with 5.6% carious teeth and partly lost restorations, while the control group had 16.7% carious teeth with partly lost restorations and 8.9% carious teeth with completely lost restorations (*p* = .004). These results would indicate SDF was beneficial for arresting caries. However, the study period was limited, and the occurrence of secondary caries was not evaluated, as radiographs were not available in the field setting where the study was conducted.

### 2.3 Variations in SDF applications

There were noticeable differences in the intervals for the SDF applications in all studies related to children (Table 4). However, SDF was applied once a year on root caries in the older population. It should be noted that the only consistent part was the concentration of SDF which was 38%.

**Table 4:** Number of studies and differences in their durations (months).

Study duration (months)	Number of studies	Percentage
36	2	18
30	1	9
24	3	27
18	1	9
12	2	18
9	1	9
6	1	9

Interestingly, the preventative fraction was 72% in Llodra et al. (2005), where SDF was applied every 6 months, while the preventative fractions were 71% and 57%, respectively, in Tan et al. (2010) and Mauro et al. (2004) where the application of the SDF was once a year. The preventative fraction observed by Llodra et al. (2005) was 31% and 15% greater than the values reported by Liu et al. (2012) and Mauro et al. (2004), respectively. It could be suggested that the optimum effect for the application of 38% SDF would be twice a year for the treatment of dental caries in permanent teeth (children and adults).

The preventative fraction of annual application of SDF in comparison with the GIC restorations and use of dental varnish containing fluoride was 45% in Mendiratta et al. (2021).

### 2.4 Assessment of bias

The assessment of bias for all studies is presented in Table 5.

**Table 5:** Assessment of bias for each study.

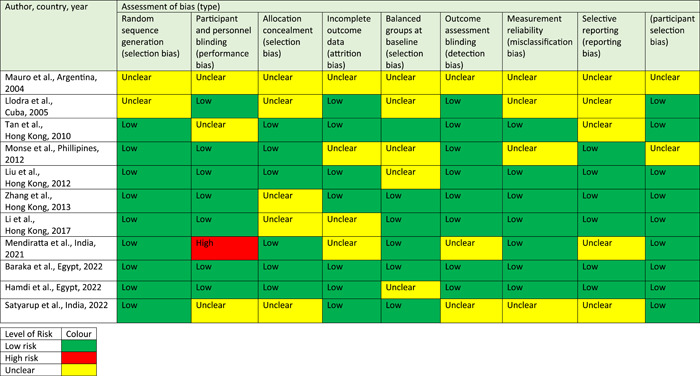

There is a lack of information available for Mauro et al. (2004) hence the risk of bias was unclear for all criteria. The generalization could give no further insight into the risk of bias for specific bias criterion subsets (Table 5).

Allocation concealment had an unclear risk of bias in a majority of the studies. Regarding Llodra et al. (2005), there was no indication regarding the allocation process for each child into different study groups. It should be noted that two previously calibrated examiners carried out the dental examinations of this study. There is evidence for higher intrareliability of an examiner when compared to inter‐reliability between examiners (Banting et al., 2011). Therefore, the measurement reliability bias was unclear. Zhang et al. (2013) reported that participants were blinded to the type of applications, and these participants were assessed at baseline and after 24 months by the same examiner. However, the evidence was unclear if the examiner was blinded to the treatment groups. Therefore, the risk of allocation concealment was unclear. Overall, there was a low risk of bias for this study (Table 5).

The risk of bias for having balanced groups at baseline was unclear for all studies related to children (Table 5). The regions where the clinical trials were conducted share some similarities, which might explain this unclear risk of bias. Llodra et al. (2005) conducted a clinical trial in the city of Santiago de Cuba, Cuba. It should be noted that there was a low fluoride content in the drinking water (0.09 ppm fluoride). In addition, it was also compulsory for children to use a 0.5% sodium fluoride oral rinse once every 2 weeks in school. Interestingly, there was widespread limited availability of fluoridated toothpaste in the city. Even though it was reported that the children were exposed to low levels of fluoride, there was no consideration in the study factoring in the bi‐weekly use of the sodium fluoride oral rinse. There was also a lack of information with regard to drop‐outs for the students. Therefore, the risk of selective reporting bias was unclear.

The clinical trial was conducted by Liu et al. (2012) in the Guangzhou province of China. There was a low fluoride content in the water system. This was the only study that added an anticipated 10% dropout rate. However, it was not clearly stated how this would affect the study. There was no explanation as to whether a 10% dropout was expected between all treatment groups or not. The duration of this study was 24 months, with the SDF application every 12 months. It was not clarified whether there would be an expected 10% dropout over the 24 months or every 12 months. In addition, the control group consisted of 128 allocated participants at baseline. The results at baseline were used to calculate *p*‐values for a number of factors in the different treatment groups, such as dental visit history and snacking habits (Liu et al., 2012). All of the *p*‐values recorded were >0.05, denoting no statistically significant differences between treatment groups and potential confounding factors. There were also *p*‐values recorded for comparing factors related to tooth surfaces (such as early caries and fissure morphology). In addition, the *p*‐values were >0.05, and there was no statistically significant difference between treatment groups. It was unclear if there was a 10% dropout calculation applied to each treatment group before the statistical tests were carried out. A lack of clarity with regard to the 19% predicted dropout was eminent. This was calculated throughout a period of 24 months that is, a 10% dropout was predicted after every application (12 months). Each different value from predicted dropout rates might have been able to influence the statistical tests to show no statistical significance, however, this was not the full picture. Therefore, the possibility of bias in the balance of individual treatment groups at baseline was unclear (Table 4).

The clinical study was carried out in two locations (Cagayan de Oro and Manila, Philippines) by Monse et al. (2012) These two cities are on opposite ends of the Philippines (north and south, respectively) where there was no mass fluoridation of water. Six schools in Cagayan de Oro and two schools in Manila were selected for this study. There was no indication if there were equal numbers of participants for each school. Furthermore, the reason for choosing these two regions was not explained. There was a lack of information on the reasons for more schools being chosen in Cagayan de Oro compared to Manila. Therefore, the risk of bias for balanced groups at baseline selection was unclear (Table 5).

The preventative range following the use of SDF was between 42%–73% in all included studies. However, the study by Zhang et al. (2013). produced extremely low values (4%–33%). The source of this difference was unclear and could not be traced in the study (Table 5).

Hamdi et al. (2022) conducted a double‐blinded randomized clinical trial. The randomization was determined by online software and implementation steps prepared opaque and well‐sealed envelopes as the assessor was not involved. The allocation for the early enamel lesions T baseline was unequal without any explanation. Participants were recruited through social network advertisements and by hanging posters in the outpatient clinic. Interestingly, there was no dropout throughout the study. Therefore, these biases were low.

Mendiratta et al. (2021) allocated participants using a sequence of random numbers. However, the authors failed to provide any further information regarding the allocation concealment and blinding processes that is, blinding of participants and assessors. Water fluoride concentration is also high in parts of Khordha and Nayagarh districts in Odisha. Satyarup et al. (2017) reported one researcher assessed the follow‐up examinations without providing any information with regard to intrareliability or interreliability. In addition, the measurement reliability was unclear. There was no explanation for dropouts, which would mean that the selective reporting bias was unclear.

The randomized controlled trial by Baraka et al. (2022) was carried out a detailed blinding and allocation processes. Allocation was concealed from the investigators, participants, and statisticians. The study provided details of dropout reasons, including changes in phone numbers and addresses; loss of contacts, and the COVID‐19 pandemic. The range of fluoride content in the tap water was 0.330–0.377 mg/L, with an average of 0.36 mg/L in Alexandria, Egypt. The outcome measurements were evaluated using clinical visual assessments and radiographs. The sample size for each group at baseline was equal. Therefore, these risks of biases were low.

Finally, Satyarup et al. (2022) performed a randomized single‐blind controlled trial in the Rohtak district of Haryana, India. The randomization and allocation concealment were conducted by a different investigator. However, blinding for the outcome assessments was unclear. The number of carious teeth at baseline for each group was equal, and the number of carious teeth for each group at follow‐up visits was different. However, there was a lack of information related to the reasons for drop‐out. Therefore, the selective reporting and incomplete outcome data bias were unclear.

## 3 DISCUSSIONS

This was the first systematic review that assessed the effect of SDF treatment on occlusal and root surfaces of permanent teeth with no age restrictions. The findings from this systematic review could only give an indication of producing similar results for studies related to children in areas with no fluoridated water and limited access to fluoridated toothpaste. The use of SDF as a treatment for dental caries in first permanent molars might not be as effective in a region with fluoridated water. This means, even if the same methodology was used in the study by Llodra et al. (2005) In Birmingham, the preventative fraction observed might be lower than what was seen in Llodra et al. (2005) This is due to the fluoridated toothpaste (Levine, 2020) and water fluoridation (Macey et al., 2018) since both of these have an effect of contributing to the caries prevention. Therefore, the results of these studies in children are limited in terms of comparison with other studies.

Li et al. (2017) and Zhang et al. (2013) included community‐dwelling elders, while, in Tan et al. (2010) study, the older people in residential homes and in Satyarup et al. (2022) trial, disabled individuals were only recruited. This should be taken into account when comparing the data sets of the four studies since the differences in results between Tan et al. (2010), Satyarup et al. (2022) and the other two studies (Satyarup et al., 2022; Zhang et al., 2013) might be related to the variations in their selection criteria. Having studies that only include older participants/disabled individuals would limit this systematic review by not allowing for speculation towards adults of other age/background groups. The three studies only assessed the effect of SDF on root caries while Satyarup et al. (2022) reported the effect of SDF use on enamel carious lesions. This was another limitation, as the SDF application was not explored in other types of dental caries.

Different durations for 11 studies were also noticed. Satyarup et al. (2022) conducted the clinical study for a period of six months while Baraka et al. (2022) had nine months only. Mauro et al. (2004) and Zhang et al. (2013) only reported results for the end of the study. The lack of data for Zhang et al. (2013) at 12 months meant that there was less comparison available with the other two studies in older adults. Tan et al. (2010) and Li et al. (2017) both reported data every 12 months, for the first 24 months.

There were variations in the control groups. Li et al. (2017) included the combination of OHI+ tonic water as the control group, whereas the other two studies in older adults used OHI+ water only. Further statistical analyses were unable to be performed due to the differences in study groups that were included in the current systematic review.

All the included studies indicated a double‐blinded design. However, the use of SDF for the treatment of dental caries in a blinded clinical trial comes with a flaw that is hard to counter. As aforementioned, SDF causes permanent black staining of the carious lesion. This would compromise the blinding procedure and potentially influence the decision‐making process.

This systematic review has limitations due to the available evidence in the literature on the use of SDF. There was a lack of studies on the effects of SDF on coronal caries in adults. In another respect, there were studies that investigated the effect of SDF on first permanent molars in children, which was promising to have a different age group. Studies on different age groups in adults for permanent teeth would be an improvement on the diversity of the current literature available. Most of the included studies assessed the specific regions that are poor and where residents lack many sources of fluoride. The effect of SDF on the treatment of dental caries in permanent teeth in regions with fluoridated water would also be an area of interest. In this respect, Mendiratta et al. (2021) reported that favorable outcomes related to SDI application. However, these results on the caries reduction could also be related to water fluoridation in that area.

Lastly, SDF causes carious lesions to turn black, due to the formation of silver oxide. If these lesions are left untreated, the staining has been shown to be irreversible. SDF provides a treatment option for carious lesions through biological and chemical means, unlike a number of other minimally invasive treatment strategies which solely rely on a chemical mode of action. In this respect, Crystal et al. (2017) assessed the parental perceptions of staining caused by the SDF. The results showed staining was deemed to be acceptable on the posterior teeth in comparison to the anterior region.

The use of potassium iodide (KI) immediately after the application of silver diamine fluoride has been shown to potentially improve the staining of carious lesions in dentine or the surrounding enamel (Patel et al., 2018). Vinh et al. (2017) also reported the use of KI immediately after applying SDF showed minimal or no staining after a period of 4 weeks in comparison to the SDF application alone.

Couple of studies investigated both the effect of SDF as well as SDF + KI when compared with tonic water alone (*p* < .001) or TCS, CPP‐ACP applications (Hamdi et al., 2022; Li et al., 2017). The application of SDF alone (62%) had a preventative fraction that was 10% greater than the preventative fraction of SDF + KI (52%). This could indicate that the addition of KI, when used in conjunction with SDF, might limit the efficacy of SDF for the arrest of carious lesions. However, the reason for these differences might totally be unrelated to the addition of KI. Both treatment groups were given oral health instructions. The treatment group of SDF only may have adhered to the instructions better than the treatment group of SDF + KI, which may have caused the difference in results. An analysis of covariance was carried out between the results for SDF and SDF + KI (*p* > .05) showing that there is a difference, however insignificant (Li et al., 2017). In addition, Hamdi et al. (2022) reported that the use of SDF‐KI arrested 54.8% of early enamel carious lesions when compared to the TCS and CPP‐ACP groups (100%) for a period of 24 months (*p* < .001). However, SDF‐KI was applied annually only, while the CPP‐ACP and TCS applications were twice a day throughout the study. The application frequency of SDI with or without KI is still unclear. There is also conflicting evidence on the level of stain reduction following the addition of KI and the efficacy of SDI plus KI on dental caries. Further research is required to assess the level of staining and efficacy on dental caries in all populations for SDF plus KI compared against SDF alone.

In summary, SDF is not commonly considered as a treatment option for dental caries in adults. Therefore, guidelines and policies need to consider including the use of SDF as part of the management of dental caries both for children and adults. However, further research is required on coronal caries in adults.

## 4 CONCLUSION

Within the limitations of this systematic review, the use of SDF is promising with high preventative fractions in children and older populations when compared to other topical applications such as dental varnish containing sodium fluoride.

## AUTHOR CONTRIBUTIONS

**Alvin Mungur**: Acquisition of data; analysis and interpretation of data; drafting the article. **Haoran Chen**: Acquisition of updated data; analysis and interpretation of data; drafting the updated results. **Saroash Shahid**: Design of the study; editing of the publication. **Aylin Baysan**: The conception and design of the study; acquisition of data; interpretation of data; editing of the article; revising it critically for important intellectual content; final approval of the version to be submitted.

## CONFLICT OF INTEREST STATEMENT

The authors declare no conflicts of interest.

## Supporting information

Supporting information.

## Data Availability

The data that support the findings of this study are available on request from the corresponding author. The data are not publicly available due to privacy or ethical restrictions.
