# Supplementary material for: A systematic review on the effect of silver diamine fluoride for management of dental caries in permanent teeth
Source: Clin Exp Dent Res. 2023 Feb 23;9(2):375–87. doi: 10.1002/cre2.716 (PMC10098297; doi:10.1002/cre2.716)
Supplement: Supplementary file 1 — Supporting information. [file CRE2-9-375-s001.docx]

**Supplementary Table. Search factors based on PICO strategy**

Boolean operators (“OR” and “AND”) were used to join search terms related to the search question

| #1 | “dental caries” OR “carious lesion” OR “enamel caries” OR “root caries” OR “coronal caries” OR “cariogenesis” OR “carious dentine” OR “carious teeth” OR “dentin caries” OR “dental decay” OR “tooth caries” OR “tooth decay” OR “proximal caries” OR “interproximal caries” OR “approximal caries” OR “occlusal caries” OR “primary caries” OR “secondary caries” OR “recurrent caries” OR “residual caries” OR “hidden caries” OR “rampant caries” OR “incipient caries” OR “early caries lesion” OR “treatment” OR “susceptibility” |
| --- | --- |
| #2 | “remineralisation” OR “remineralization” OR “caries arrest” OR “arresting dentine caries” OR “arresting root caries” OR “arresting dental caries” OR “arrest root caries” OR “arrest dental caries” OR “arrest dentine caries” OR “remineralising effect” OR “remineralizing effect” OR “arresting effect” OR “caries management” OR “caries control” OR “arresting approximal caries” OR “arresting occlusal caries” OR “arresting proximal caries” OR “arresting tooth decay” OR “arresting tooth caries” OR “arresting interproximal caries” OR “arresting primary caries” OR “arresting secondary caries” OR “arresting recurrent caries” OR “arresting residual caries” OR “arresting hidden caries” OR “arresting rampant caries” OR “arresting coronal caries” OR “arresting dental decay” OR “arresting root decay” OR “arresting dentine decay” OR “arresting dentin decay” OR “arresting incipient caries” OR “arresting early carious lesion” OR “arrest approximal caries” OR “arrest occlusal caries” OR “arrest proximal caries” OR “arrest tooth caries” OR “arrest interproximal caries” OR “arrest primary caries” OR “arrest secondary caries” OR “arrest recurrent caries” OR “arrest residual caries” OR “arrest hidden caries” OR “arrest rampant caries” OR “arrest coronal caries” OR “arrest coronal caries” OR “arrest dental decay” Or “arrest root decay” Or “arrest dentine decay” OR “arrest dentin decay” OR “arrest incipient caries” OR “arrest early carious lesion” OR “arresting” OR “remineralising” OR “remineralise incipient caries” OR “remineralise dentine caries” OR “remineralise root caries” OR “remineralise dentin caries” OR “remineralise dental caries” OR “remineralise tooth caries” OR “remineralise approximal caries” OR “remineralise occlusal caries” OR “remineralise proximal caries” OR “remineralise tooth decay” OR “remineralise interproximal caries” OR “remineralise primary caries” OR “remineralise secondary caries” OR “remineralise recurrent caries” OR “remineralise residual caries” OR “remineralise hidden caries” OR “remineralise rampant caries” OR “remineralise coronal caries” OR “remineralise dental decay” OR “remineralise root decay” OR “remineralise dentin decay” OR “remineralise dentine decay” OR “remineralise early carious lesion” OR “remineralize incipient caries” OR “remineralize dentine caries” OR “remineralize root caries” OR “remineralize dentin caries” OR “remineralize dental caries” OR “remineralize tooth caries” OR “remineralize approximal caries” OR “remineralize occlusal caries” OR “remineralize proximal caries” OR “remineralize tooth decay” OR “remineralize interproximal caries” OR “remineralize primary caries” OR “remineralize secondary caries” OR “remineralize recurrent caries” OR “remineralize residual caries” OR “remineralize hidden caries” OR “remineralize rampant caries” OR “remineralize coronal caries” OR “remineralize dental decay” OR “remineralize root decay” OR “remineralize dentin decay” OR “remineralize dentine decay” OR “remineralize early carious lesion” OR “incipient caries remineralisation” OR “dentine caries remineralisation” OR “root caries remineralisation” OR “dentin caries remineralisation” OR “dental caries remineralisation” OR “tooth caries remineralisation” OR “approximal caries remineralisation” OR “occlusal caries remineralisation” OR “proximal caries remineralisation” OR “tooth decay remineralisation” OR “interproximal caries remineralisation” OR “primary caries remineralisation” OR “secondary caries remineralisation” OR “recurrent caries remineralisation” OR “residual caries remineralisation” OR “hidden caries remineralisation” OR “rampant caries remineralisation” OR “coronal caries remineralisation” OR “dental decay remineralisation” OR “root decay remineralisation” OR “dentin decay remineralisation” OR “dentine decay remineralisation” OR “early carious lesion remineralisation” OR “incipient caries remineralization” OR “dentine caries remineralization” OR “root caries remineralization” OR “dentin caries remineralization” OR “dental caries remineralization” OR “tooth caries remineralization” OR “approximal caries remineralization” OR “occlusal caries remineralization” OR “proximal caries remineralization” OR “tooth decay remineralization” OR “interproximal caries remineralization” OR “primary caries remineralization” OR “secondary caries remineralization” OR “recurrent caries remineralization” OR “residual caries remineralization” OR “hidden caries remineralization” OR “rampant caries remineralization” OR “coronal caries remineralization” OR “dental decay remineralization” OR “root decay remineralization” OR “dentin decay remineralization” OR “dentine decay remineralization” OR “early carious lesion remineralization” OR “remineralising efficacy” OR “remineralizing efficacy” OR “remineralisation effect” OR “remineralization effect” OR “antibacterial” OR “caries reversal” OR “reversal of caries” |
| #3 | “sdf varnish” OR “silver diamine fluoride varnish” OR “silver diamine fluoride varnish” OR “topical sdf” OR “topical silver diamine fluoride” OR “topical silver diamine fluoride” OR “fluoride varnish” OR “topical fluoride” OR “silver diammine fluoride” OR “SDF” OR “silver diamine fluoride” OR “varnish” OR “fluoride” OR “topical fluoride agent” OR “topical silver diamine fluoride agent” OR “topical silver diammine fluoride agent” OR “topical sdf agent” OR “fluoridated varnish” OR “fluoridated topical agent” OR “sodium fluoride varnish” OR “topical sodium fluoride” OR “topical sodium fluoride agent” OR “NaF” OR “NaF varnish” OR “topical NaF” OR “topical NaF varnish” OR “fluoride solution” OR “NaF solution” OR “sdf solution” OR “silver diamine fluoride solution” OR “silver diamine fluoride solution” |
| #4 | “adult teeth” OR “adults” OR “permanent dentition” OR “older adults” OR “permanent teeth” OR “secondary teeth” OR “secondary dentition” OR “elderly” OR “care homes” “permanent molars” OR “permanent first molars” OR “secondary molars” OR “adolescents” OR “schoolchildren” OR “children” OR “child” |
| Search #1 AND #2 AND #3 AND #4 | |
| **Boolean operators (“OR” and “AND”) were used to join search terms** | |
